# Genetic diversity and forensic application of Y-filer STRs in four major ethnic groups of Pakistan

**DOI:** 10.1186/s12864-022-09028-z

**Published:** 2022-11-30

**Authors:** Muhammad Salman Ikram, Tahir Mehmood, Allah Rakha, Sareen Akhtar, Muhammad Imran Mahmood Khan, Wedad Saeed Al-Qahtani, Fatmah Ahmed Safhi, Sibte Hadi, Chuan-Chao Wang, Atif Adnan

**Affiliations:** 1 Department of Anthropology and Ethnology, Institute of Anthropology, School of Sociology and Anthropology, Xiamen University, Xiamen, China; 2 Institute of Chemistry, University of Sargodha, Sargodha, 40100 Punjab Pakistan; 3 Centre for Applied and Molecular Biology (CAMB), University of the Punjab, Lahore, 53700 Punjab Pakistan; 4 Department of Forensic Sciences, University of Health Sciences, Lahore, 54600 Pakistan; 5 International Committee of Red Cross, Markaz G 11, Islamabad, Pakistan; 6 Department of Forensic Sciences, College of Criminal Justice, Naïf Arab University of Security Sciences, Riyadh, 11452 Kingdom of Saudi Arabia; 7 Department of Biology, College of Science, Princess Nourah bint Abdulrahman University, P.O. Box 84428, Riyadh, 11671 Saudi Arabia

**Keywords:** Punjabi, Sindhi, Baloch, Pathan, Yfiler amplification kit, Forensic and population genetic

## Abstract

17 Y-chromosomal STRs which are part of the Yfiler Amplification Kit were investigated in 493 unrelated Pakistani individuals belonging to the Punjabi, Sindhi, Baloch, and Pathan ethnic groups. We have assessed the forensic parameters and population genetic structure for each group. Among the 493 unrelated individuals from four ethnic groups (128 Baloch, 122 Pathan, 108 Punjabi, and 135 Sindhi), 82 haplotypes were observed with haplotype diversity (HD) of 0.9906 in Baloch, 102 haplotypes with HD value of 0.9957 in Pathans, 80 haplotypes with HD value of 0.9924 in Punjabi, and 105 haplotypes with HD value of 0.9945 in the Sindhi population. The overall gene diversity for Baloch, Pathan, Punjabi, and Sindhi populations was 0.6367, 0.6479, 0.6657, and 0.6112, respectively. The results had shown us that Pakistani populations do not have a unique set of genes but share the genetic affinity with regional (Central Asia and Northern India) populations. The observed low gene diversity (heterozygosity) values may be because of endogamy trends and this observation is equally supported by the results of forensic parameters which are mostly static across 4 combinations (minimal STRs, extended 11 Y-STRs, Powerplex 12 Y System, and Yfiler 17 Y-STRs) of STRs in these four populations.

**Supplementary Information:**

The online version contains supplementary material available at 10.1186/s12864-022-09028-z.

## Introduction

The genetic makeup of Pakistan’s various ethnic groups was forged by successive waves of immigration from Central Asia and South Asia since the end of the last Ice Age. Throughout its long ancient history, the Indus Valley has been known for welcoming different people, faiths, and cultures. The Indus was a region where early human ancestors encountered soon after they left Africa between 50,000 to 70,000 years ago. Evidence of these early humans can be found throughout Pakistan today at Soan, Rawat, Makli Hill, Bajaur, and Sanghao. Approximately 9000 years ago they began establishing cities such as Mehrgarh, which eventually expanded to represent the Harappan culture (Indus Valley Civilization) in 3000 BCE (Before the Common Era), rivaling the early city-states of Mesopotamia. Harappans fused culturally with the Aryans, forming Indo Aryans and Indo Iranians, which today culminates in the native ethnic groups of Pakistan. It was through these various influences by Pakistani ethnic groups would be forged into its multi-ethnic society today [1].

Pakistanis are divided genetically into 11 major distinct groups: Baloch, Brahui, Burusho, Hazara, Kalash, Kashmiri, Makrani, Parsi, Pashtun, Punjabi, and Sindhi [2]. The uniparental marker studies (mtDNA) showed that these ethnic groups share most of their maternal ancestry with South Asians Eurasians East Asians, West Asians, or Sub-Saharan Africans [3–8].

Most of these studies focused on the control region sequencing of mtDNA. A limited number of studies are available related to Y Chromosomal analysis in Pakistani ethnic groups and most of their focus was only on allelic frequency analysis along with basic forensic parameters [9–12].

In population genetics, the non-recombining region of the human Y chromosome (NRY) has attracted much attention for its unique inheritance characteristics [12, 13]. The phenomenon of mutation can be observed much faster on Y chromosomal short tandem repeats (Y-STRs) as compared to Y-SNPs (3.78 × 10^− 4^ to 7.44 × 10^− 2^) [11, 14], and they are used in evolutionary and genealogical studies to measure the historically distinct incidences [15, 16], regardless of time scale and size of mutations. Y-STRs are commonly employed in forensic casework to characterize male contributions to mixed male-female biological materials, notably in sexual assault instances [17], and paternity cases involving male offspring, particularly in deficiency paternity cases where the putative father is unavailable and replaced by one of his male relatives.

In the present study, we planned to assess the forensic parameters and genetic structure of four major ethnic groups from Pakistan on Y chromosomal STRs. For this, we have investigated four main ethnic groups (Punjabi, Sindhi, Pathan, and Balochi) of Pakistan using AmpFlSTR Y-filer PCR Amplification Kit (Life Technologies). We also gathered 17 commonly used Y-STR loci data which is available at YHRD (Y chromosomal Haplotype reference database) [18]. We calculated and compared forensic diversity indices and explored the genetic variance between these ethnic groups.

## Materials and methods

### Samples used in the study

Blood samples were collected from a total of 493 unrelated individuals, who are residents of respective provinces for at least three generations (128 Baloch, 122 Pathan, 108 Punjabi, and 135 Sindhi) across four provinces (Baluchistan, Khyber Pakhtunkhwa, Punjab, and Sindh) of Pakistan. All participants gave their informed consent in writing after the study aims and procedures were carefully explained to them. The study was approved by the ethical review board of the University of Sargodha, Sargodha Punjab, Pakistan, and in accordance with the standards of the Declaration of Helsinki 1964.

### DNA extraction

All blood samples were stored at − 20 °C before DNA extraction. DNA was isolated using the *ReliaPrep™ Blood gDNA Miniprep System* (Promega, Madison, USA) according to the manufacturer’s instructions. The quantities of extracted DNA samples were determined using a NanoDrop spectrophotometer (Thermo Scientific, Wilmington DE, USA). These samples were diluted accordingly to make a final concentration of 2 ng/μl.

### PCR amplification and Y-STR typing

Diluted DNA samples were genotyped at 17 Y-STRs using the AmpFlSTR Yfiler™ kit (Thermo Fisher Scientific) according to the manufacturer’s instructions. PCR amplification was carried out using the Applied Biosystems® GeneAmp® PCR System 9700 thermal cyclers. AmpFlSTR Y-filer (Thermo Fisher Scientific) PCR amplifications were performed as recommended by the manufacturer, although using half of the recommended reaction volume (12.5 μl). Subsequently, separation and detection were performed using an Applied Biosystems™ 3500 Series Genetic Analyzer (Life Technologies). Internal controls (negative and the 9947A DNA positive control) were genotyped along with each batch of samples to ensure that the results were reproducible and accurate. Finally, the raw data were analyzed using GeneMapper *ID* v4.1 software (Life Technologies). We strictly followed the recommendations of the DNA Commission of the International Society of Forensic Genetics (ISFG) on the analysis of Y-STRs [19].

### Statistical analyses

Haplotype and allelic frequencies of these four ethnic groups (Baloch, Pathan, Punjabi, and Sindhi) were calculated using the direct counting method. Gene diversity (GD), haplotype diversity (HD), and discrimination capacity (DC) were calculated using the following formulas:

$$\begin{array}{c}\text{GD}=\frac n{n-1}\left(\Sigma p_{ai}^2\right)\\\begin{array}{c}\mathrm{HD}=\frac n{n-1}\left(\Sigma p_{hi}^2\right)\\\text{MP}=\Sigma p_{hi}^2\end{array}\end{array}$$

Genetic distances between these four ethnic groups and reference population analysis of molecular variance (AMOVA) and multidimensional scaling (MDS) that exploit variations among populations were performed using YHRD online tools (http://www.yhrd.org) based on pairwise R*st* and F*st* values. Reduced dimensionality spatial representation of the populations based on Rst values, was performed using multi-dimensional scaling (MDS) with IBM SPSS Statistics for Windows, Version 23.0 (IBM Corp., Armonk, NY, USA). A neighbor-joining phylogenetic tree was constructed for these four ethnic groups and the reference populations based on a distance matrix of F*st* using the Mega7 software [20]. We also predicted Y-SNP haplogroups in the samples from Y-STR haplotypes using the Y-DNA Haplogroup Predictor NEVGEN (http://www.nevgen.org). Using the program Network 4.1.1.2., the median-joining network was constructed from data of these four ethnic groups for 14 Y-STRs (DYS19, DYS389II-I, DYS390, DYS391, DYS392, DYS393, DYS437, DYS438, DYS439, DYS448, DYS456, DYS458, DYS635, Y_GATA_H4).

## Results and discussion

### Allelic frequency and forensic parameters

**Fig. 1:**
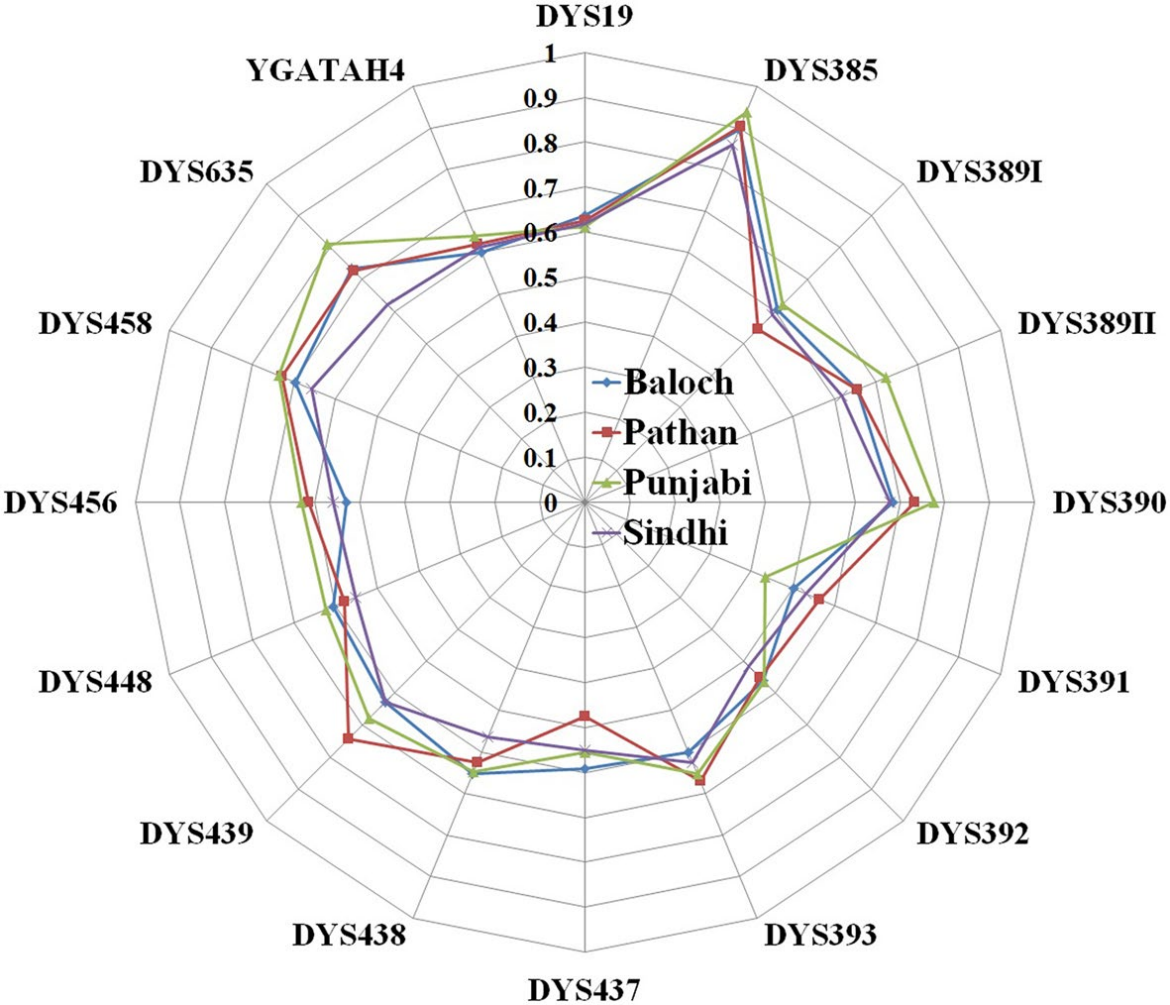
Heterozygosity scattered plot for four populations

Successfully generated genotypes at 17 Y-STRs from 493 male individuals (128 Baloch, 122 Pathan, 108 Punjabi, and 135 Sindhi) across four provinces (Balouchistan, Khyber Pakhtunkhwa, Punjab, and Sindh) of Pakistan are summarized in Table S1. Haplotype data is already made accessible via the Y-chromosome Haplotype Reference Database (YHRD) under accession numbers YA004595, YA004626, YA003905, and YA004625 for Baloch, Pathan, Punjabi, and Sindhi, respectively. Allelic frequencies ranged from 0.0078 to 0.6967 across four ethnic groups. Allele numbers or combinations ranged from 3 (DYS389I) to 24 (DYS385) for the Baloch population, 3 (DYS389I) to 31 (DYS385) for the Pathan population, 3 (DYS389I, DYS391, and YGATAH4) to 30 (DYS385) for the Punjabi population and 3 (DYS389I and DYS438) to 21(DYS385) for Sindhi population (Table S2). The locus diversity (GD) ranged from 0.5017 (DYS391) to 0.8967 (DYS385) for Baloch population, 0.4767 (DYS437) to 0.9040 (DYS385) for Pathan population, 0.4339 (DYS391) to 0.9382 (DYS385) for Punjabi population and 0.5151 (DYS392) to 0.8586 (DYS385) for Sindhi population **(**Fig. 1**)**. Other forensic parameters such as polymorphic information content (PIC), matching probability (MP), and discrimination probability (DP) showed the same trends as we have observed for locus diversities (GD).

We assessed the haplotype resolution at four levels **(**Table 1**)**, the minimal 9 Y-STRs loci (MH-9), the extended 11 Y-STRs loci (SWGDAM-11), PowerPlex Y12 STRs loci (PPY-12), and Y-filer 17 STRs loci (Yfiler-17). A total of 82 haplotypes were observed at Y-filer 17 STRs loci with haplotype diversity (HD) 0.9906 and discriminatory capacity (DC) 0.6250 while among these 82 haplotypes 40.62% (52) were unique with random matching probability (RMP) 0.0171 for the Baloch population. When the number of STRs was reduced from 17 to 12 (PPY-12), we did not observe much change in the values of these forensic parameters. In the Pathan population, at Y-filer 17 STRs loci we have observed 102 haplotypes with haplotype diversity of 0.9957, a discrimination capacity of 0.8360, among these 102 haplotypes 73.77% (90) were unique with a random matching probability of 0.0125. After reducing the number of STRs to 12, 11, and 9 we did observe any change in any of these forensic parameters. In the Punjabi population, at Y-filer 17 STRs loci we have observed 80 haplotypes with haplotype diversity of 0.9924, a discrimination capacity of 0.7407, among these 80 haplotypes 58.33% (63) were unique with a random matching probability of 0.0168. When we reduced the number of STRs to 12, 11 and 9 number of haplotypes also reduced to 76, 76, and 75, respectively. In the Sindhi population, we have observed a static trend across 4 combinations of STRs. We have observed 105 haplotypes with haplotype diversity of 0.9945, a discrimination capacity of 0.7777, among these 105 haplotypes 65.92% (89) were unique with a random matching probability of 0.0129. The overall gene diversity for Baloch, Pathan, Punjabi, and Sindhi populations was 0.6367, 0.6479, 0.6657, and 0.6112, respectively. These low gene diversity (heterozygosity) values showed that these populations are endogamous and this observation is equally supported by the results of forensic parameters which are mostly static across 4 combinations of STRs in these four populations.

**Table 1:** Forensic parameters of four Pakistani populations (Baloch, Pathan, Punjab, and Sindhi) at 4 levels

	9 Y-STRs	11 Y-STRs	PP 12 Y-STRs	Yfiler 17 STRs
**Baloch**				
*Total Samples*	128	128	128	128
*RMP*	0.0186	0.0186	0.0186	0.0171
*HD*	0.9892	0.9892	0.9892	0.9906
*TH*	80	80	80	82
*UH*	52	52	52	52
*DC*	0.625	0.625	0.625	0.625
*% of UH*	40.62%	40.62%	40.62%	40.62%
**Pathan**				
*Total Samples*	122	122	122	122
*RMP*	0.0125	0.0125	0.0125	0.0125
*HD*	0.9957	0.9957	0.9957	0.9957
*TH*	102	102	102	102
*UH*	90	90	90	90
*DC*	0.836	0.836	0.836	0.836
*% of UH*	73.77%	73.77%	73.77%	73.77%
**Punjabi**				
*Total Samples*	108	108	108	108
*RMP*	0.0195	0.0182	0.0182	0.0168
*HD*	0.9896	0.991	0.991	0.9924
*TH*	75	76	76	80
*UH*	58	58	58	63
*DC*	0.6944	0.7037	0.7037	0.7407
*% of UH*	53.70%	53.70%	53.70%	58.33%
**Sindhi**				
*Total Samples*	135	135	135	135
*RMP*	0.0129	0.0129	0.0129	0.0129
*HD*	0.9945	0.9945	0.9945	0.9945
*TH*	105	105	105	105
*UH*	89	89	89	89
*DC*	0.7777	0.7777	0.7777	0.7777
*% of UH*	65.92%	65.92%	65.92%	65.92%

***RMP*** Random matching probability, ***HD*** Haplotype diversity, ***TH*** Total haplotypes, ***UH*** Unique haplotypes, ***DC*** Discrimination capacity

### Genetic relationship between current and previous studied Pakistani population

**Fig. 2:**
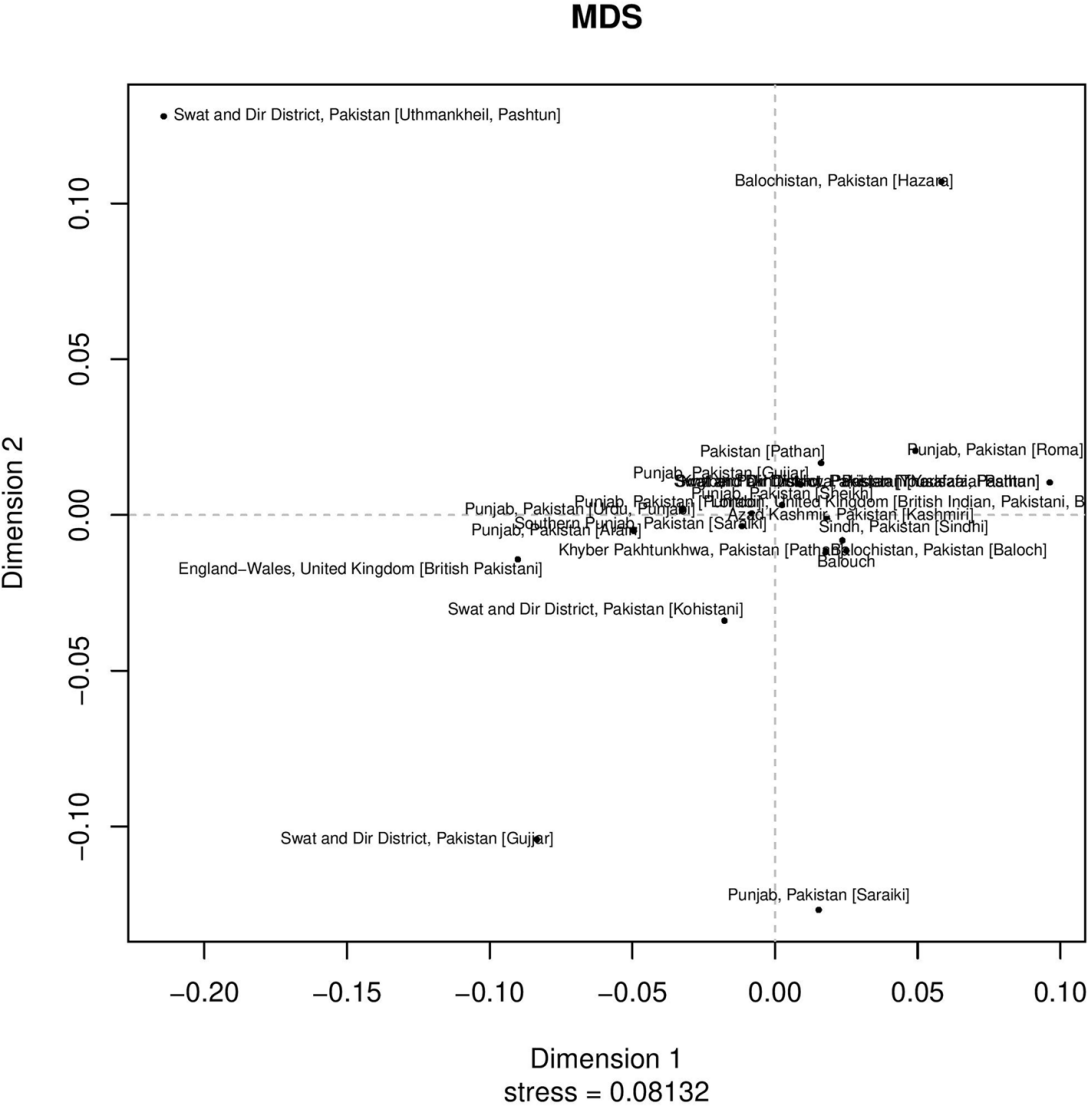
Two-dimensional plot from multi-dimensional scaling analysis of R*st*-values based on Yfiler haplotypes for the currently studied four populations with other reference populations from Pakistan

**Fig. 3:**
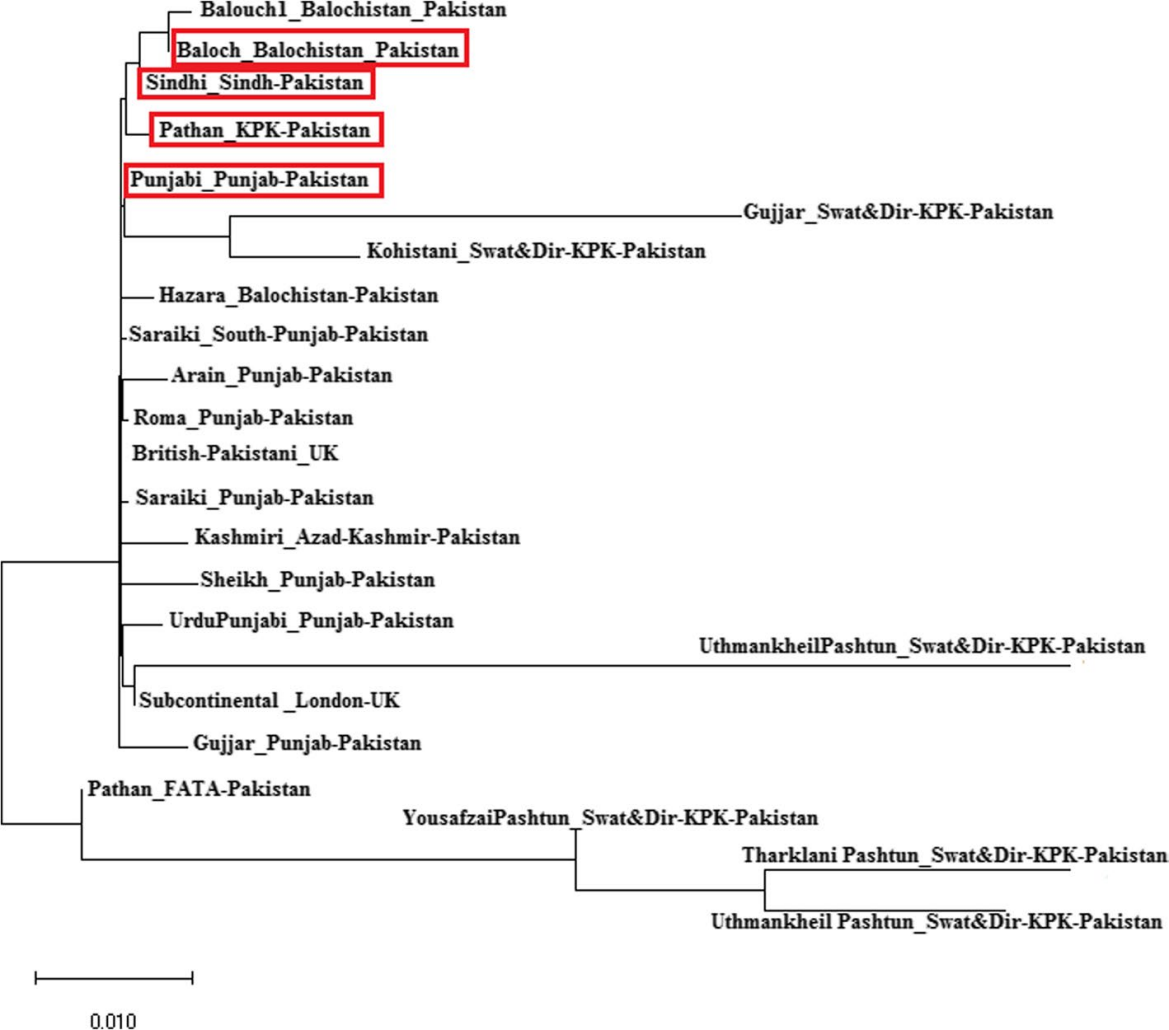
Neighbor-joining tree based on the F*st* values based on Yfiler haplotypes for the currently studied four populations with other reference populations from Pakistan

Most of the Pakistani ethnic groups are thought to have a blend of Central Asian and European ancestors [2]. Utilizing the overlapping 17 Y-STRs loci, we estimated Rst values between currently studied four Pakistani ethnic groups and previously studied Pakistani ethnic groups [15, 16, 21–23], and MDS plot was utilized to display the results. **(**Fig. 2**)**. The majority of Pakistani ethnic groups were located in the middle of the MDS plot, except for the Uthmankheil, Pashtun, Hazara, Saraki, and Gujjar populations, who were located on the plot’s boundaries. Among 23 Pakistani populations (Table S3) previously studied Baloch population (0.0033) from Baluchistan, Pakistan showed the closest distance which was followed by the Pathan population (0.0058) from Khyber Pakhtunkhaw, Pakistan while Uthmankheil, Pashtun (0.3247), Gujjar population (0.1541) from KPK showed the greatest genetic distance from the Baloch population. Evolutionary relationships among Pakistani populations were inferred from the Neighbor-joining tree based on F_*ST*_ values **(**Fig. 3**).** In neighbor-joining trees, usually, an admixed population will always lie on the path between the source populations [24]. According to Fst values (Table S4), the Tharklani Pashtun population (0.0788) from Swat and Dir district from Khyber Pakhtunkhaw, Pakistan showed the greatest distance followed by Yousafzai Pashtun (0.0765) population from Swat and Dir district from Khyber Pakhtunkhaw, Pakistan while Baloch population (− 0.0035) from Baluchistan, Pakistan showed the closest distance with Balochi population.

### Genetic relationship with regional populations

**Fig. 4:**
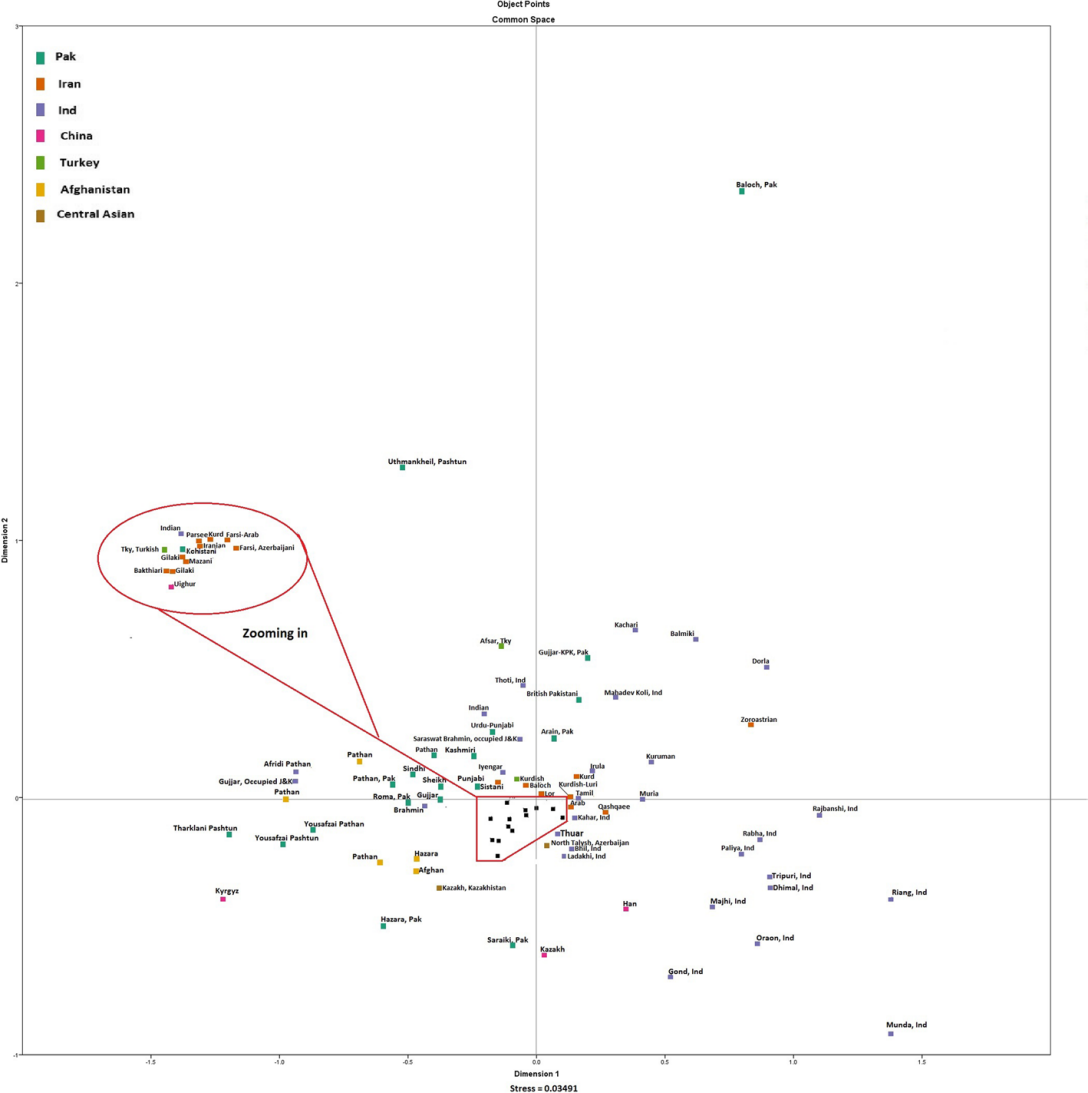
Two-dimensional plot from multi-dimensional scaling analysis of R*st*-values based on Yfiler haplotypes for the currently studied four populations with other regional populations

**Fig. 5:**
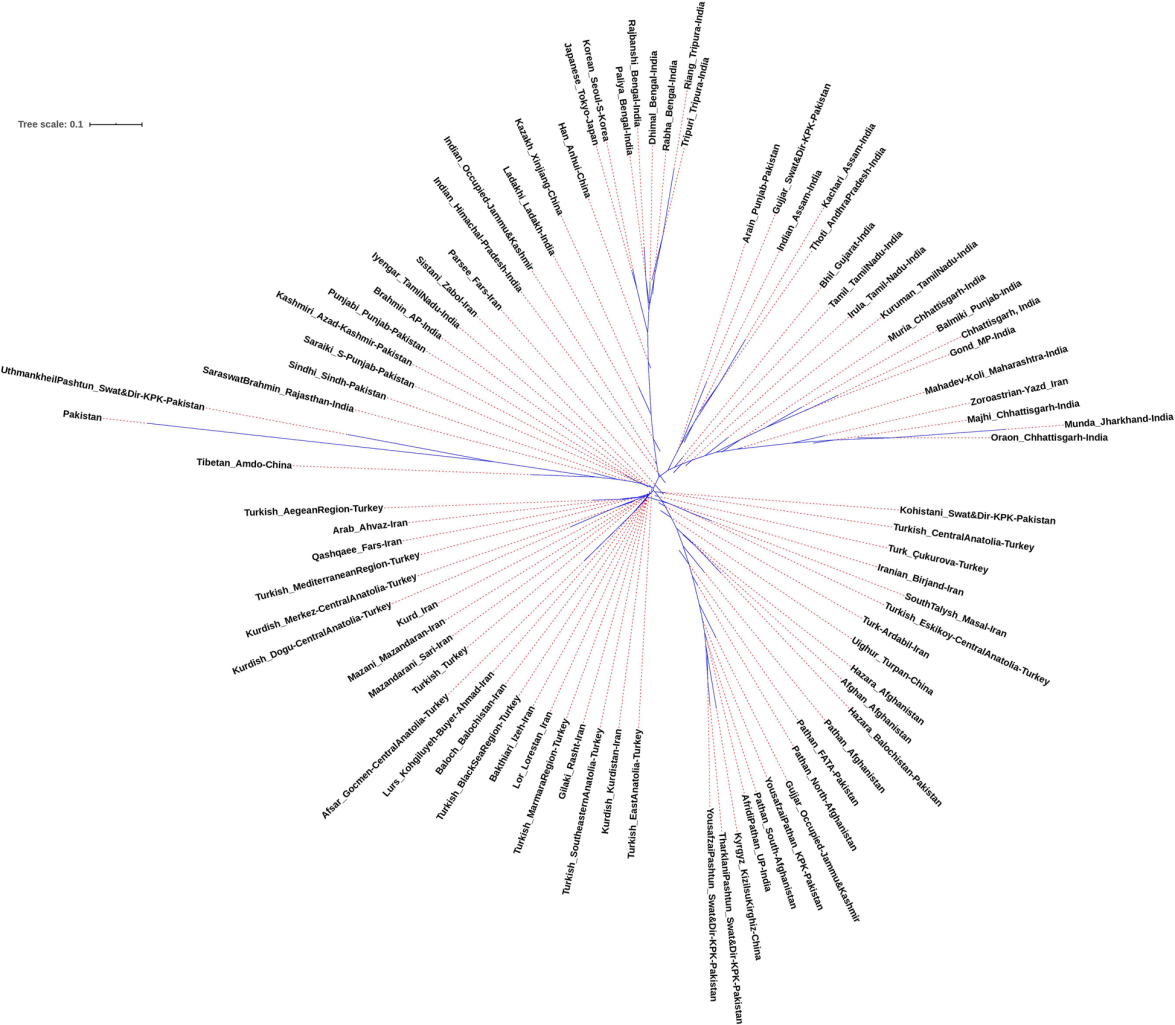
Neighbor-joining tree based on the F*st* values based on Yfiler haplotypes for the currently studied four populations with other regional populations

We compared these four populations with other regional populations from Afghanistan, China, Central Asia, India, Iran, and Turkey. The majority of Pakistani ethnic groups were placed along with Afghani, Central Asian, Iranian, and Turkic ethnicities on the left side of the MDS plot **(**Fig. 4**)**. The genetic distances (Rst) between the Punjabi population and other reference are summarized in (Table S5). Punjabi population was most closely related to the Lurs population (− 0.0064) from Kohgiluyeh-Buyer Ahmad, Iran followed by the Saraiki population (0.0015) from Southern Punjab, Pakistan while the Kazakh population (0.4081) from Altai, Xinjiang, China was most distantly related followed by Kyrgyz population (0.2355) from Kizilsu Kirghiz, China. Our results related to these four populations are consistent with our hypothesis that most the Pakistani populations have a gene pool derived from Central Asia and European populations. Modern-day Pakistan was the main gateway to India and thus Pakistani populations are mosaic of European and Central Asian populations. Evolutionary relationships among Pakistani populations and other regional reference populations were inferred from the Neighbor-joining tree based on F_*ST*_ values **(**Fig. 5**)**. Punjabi population showed genetic association with Baloch, Balochistan, Pakistan (0.0028) followed by the Iranian population from Iran (0.0038) while the Kazakh population from Altai, Xinjiang, China (0.0805) and Kazakh population from East Kazakhstan, Kazakhstan (0.1808) (Table S6).

#### Ancestry information of Pakistani ethnic groups using Y-STRs

**Fig. 6:**
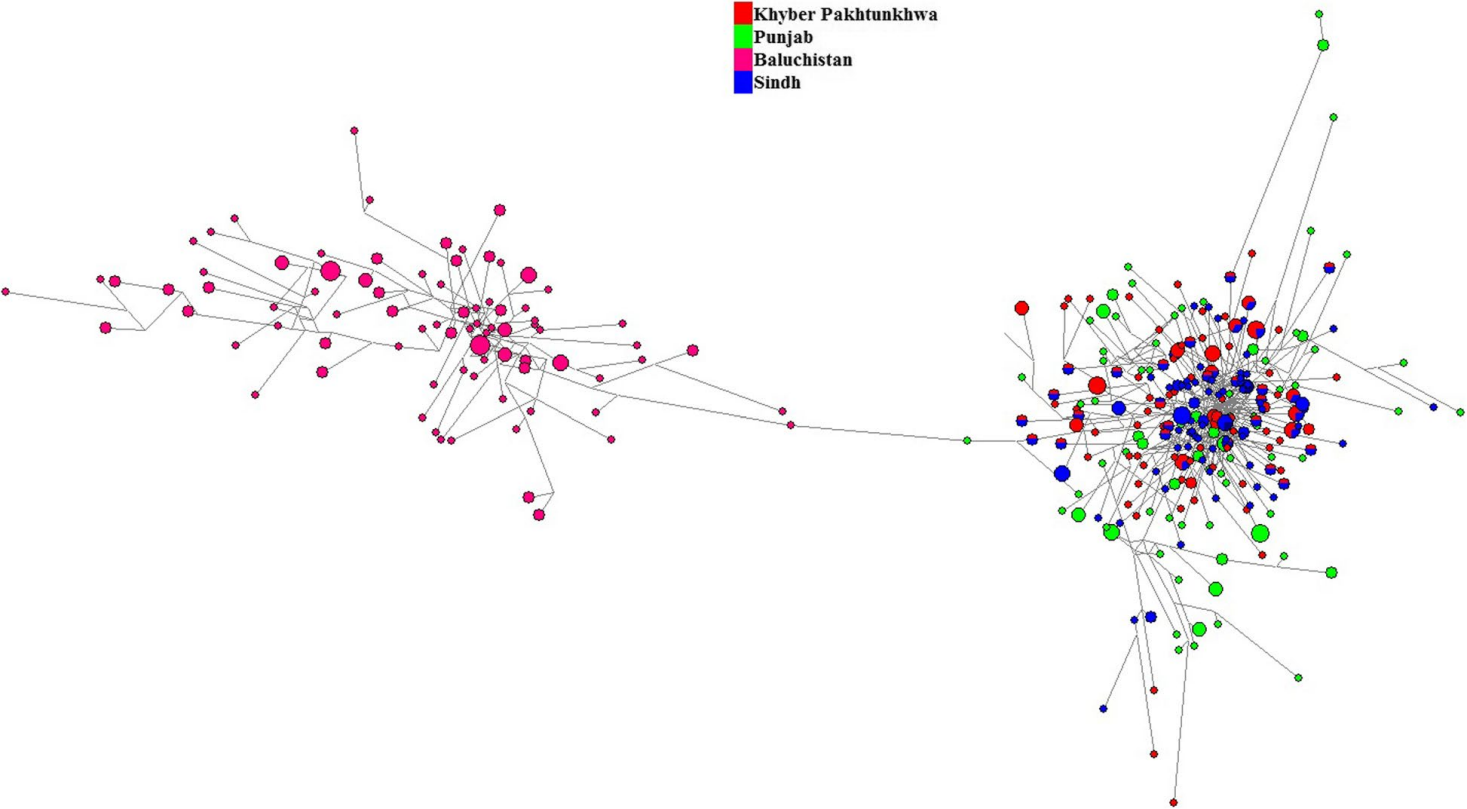
The median-joining network for four populations based on 14 Y-STRs

**Fig. 7:**
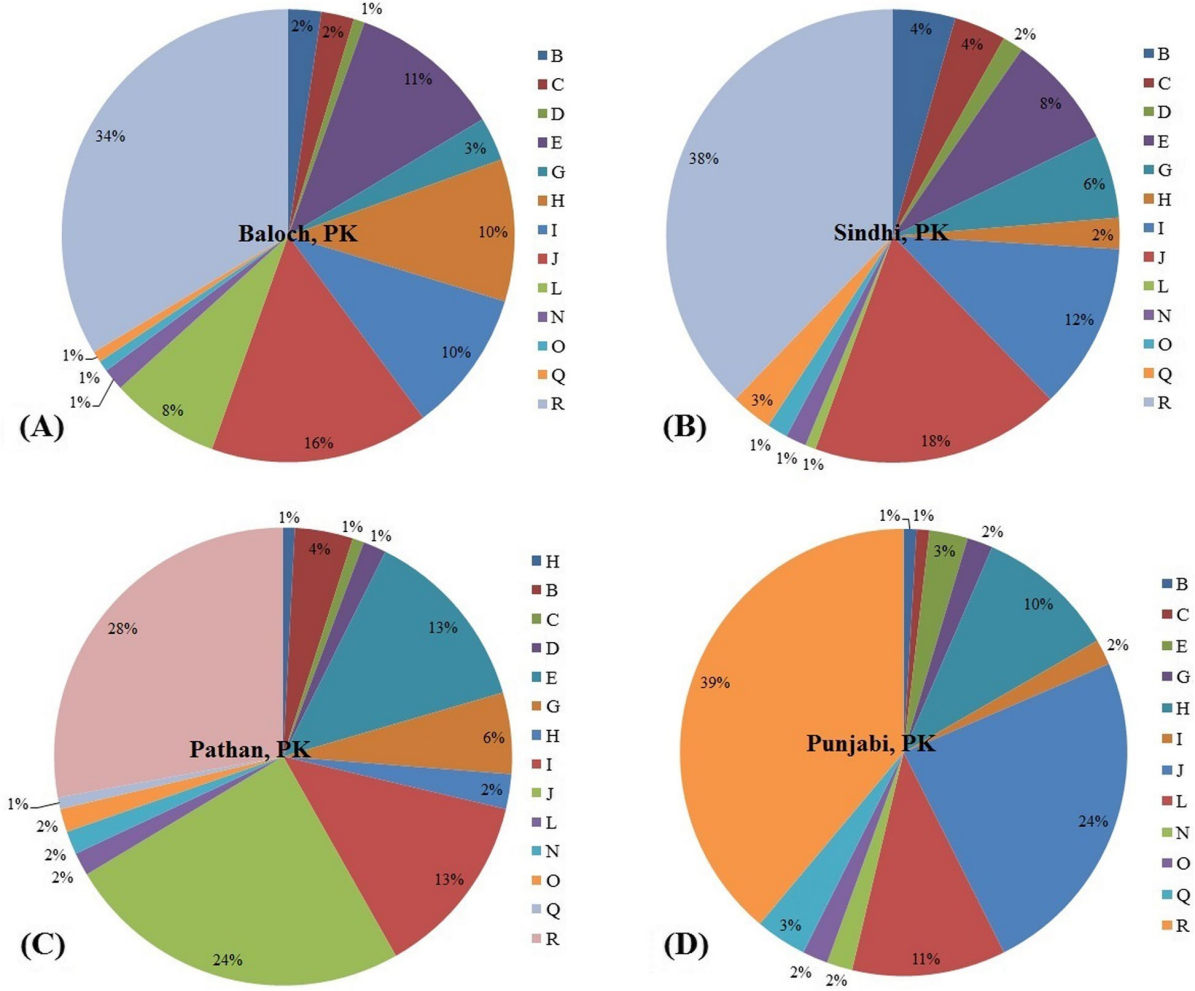
A stacked histogram with haplogroup composition for these four populations

Ethnic groups which are situated in Punjab province (Saraki, Punjabi, etc.) are admixture populations and determining their ancestry is challenging because of their admixture nature. Information about ancestry plays an important role in forensic genetic investigations. So we have to use NEVGEN software to calculate haplogroups from STRs. Only Six haplogroups (E, H, I, J, L, and R) have accounted for 84% of these samples among 4 major ethnic groups from Pakistan. The median-joining network of haplotypes **(**Fig. 6**)** showed the bulk of R haplogroup. We also presented a stacked histogram with the haplogroup composition of these populations in Fig. 7**.**

##### Haplogroup E (9%)

Haplogroup E is 9% of currently studied populations and is the most frequent haplogroup in West Asia and East Africa [25, 26]. This haplogroup originated around 65KYA (thousand years ago) [27]. The frequency of this haplogroup in Punjabi Sindhi, Pathan, and Baloch populations was 3, 8, 13, and 11%, respectively.

##### Haplogroup H (6%)

Haplogroup H is 6% of currently studied populations and is the most frequent haplogroup in South Indians and Roma people. It also originated in 48,5KYA in the south and west Asia [28]. The frequency of this haplogroup in Punjabi, Sindhi, Pathan, and Baloch populations was 10, 8, 13, and 11%., respectively.

##### Haplogroup I (9%)

Haplogroup I is 9% of the currently studied population. Subclades I1 and I2 are found in the majority of modern European people, with maxima in Northern and Southeastern European nations. Haplogroup I appear to have evolved in Europe, as evidenced by its presence in Palaeolithic sites across the continent [29], but not elsewhere. It split from its common ancestor IJ* some 43,000 years ago [30]. The frequency of this haplogroup in Punjabi, Sindhi, Pathan, and Baloch populations was 2, 12,13, and 10%., respectively.

##### Haplogroup J (20%)

Haplogroup J accounts for 20% of currently studied populations and this haplogroup is predominately found in Arabian Peninsula. The origin of this haplogroup is from the Middle East area known as the Fertile Crescent, comprising the Palestine, Jordon, Syria, Lebanon, and Iraq 42,9KYA [31]. This haplogroup was transmitted to the Subcontinent by merchants from the Arabian Peninsula [32]. The frequency of this haplogroup is 24, 18, 24, and 16% in Punjabi, Sindhi, Pathan, and Baloch populations, respectively.

##### Haplogroup L (5%)

Haplogroup L accounts for 5% of currently studied populations and this haplogroup is believed to have originated in the Middle East or Sub-continent 25-30KYA [33]. The spread of this haplogroup was distributed mainly because of trade between Arabian Peninsula and Sub-continent. The frequency of this haplogroup in Punjabi, Sindhi, Pathan, and Baloch populations was 11, 1, 2, and 8%., respectively.

##### Haplogroup R (35%)

This is the dominating haplogroup in Pakistani populations. Haplogroup R originated in the north of Asia about 27KYA years ago (ISOGG, 2017). It is the most frequent haplogroup in Europe and Russia and in some parts it is 80% of the population. Some believes its one branch originated in the Kurgan culture and their people were responsible for the taming of the horses and speaks the Indo-European languages [34]. The frequency of this haplogroup in Punjabi, Sindhi, Pathan, and Baloch populations was 39, 38, 28, and 34%., respectively.

### Languages and genetic diversity

Pakistan is a diverse nation where several different languages are used as first languages [35, 36]. The bulk of Pakistan’s languages are from the Indo-Iranian branch of the Indo-European language family [37, 38]. Urdu is Pakistan’s national language while it shares official status with English and it is the preferred and dominant language used for inter-ethnic communication [36]. Pakistan’s numerous ethno-linguistic groups speak a variety of regional languages as first languages. Punjabi, Pashto, Sindhi, Saraiki, Urdu, Balochi, Hindko, Pahari-Pothwari, and Brahui are among the languages with over a million speakers apiece [35, 37–39]. Although genetic differences can be linked to cultural, linguistic, and geographical differences, it is sometimes impossible to separate the individual effects of these elements since culture, language, and geography are all linked. Individual impacts must be distinguished by an informative genetic system and populations in which culture, language, and geography are not coupled [40] but Pakistani populations supply this evidence. Based on Y chromosomal analysis, Pakistani languages such as Balochi, Punjabi, Pushto, and Sindhi are from the Indo-Iranian branch of the Indo-European language family [37, 38] which are predominantly spoken in Balochistan, Punjab, Khyber Pakhtunkhwa, and Sindh, respectively. These languages demonstrate the genetic diversity in these populations. Punjabi and Sindhi languages are also spoken in Northern Indian regions such as Punjab, Jammu, and Kashmir, Himachal Pradesh, Haryana, and Rajasthan. and Punjabi and these populations showed more genetic affinity with Northern Indian populations. Balochi, Persian and Pushto languages are also spoken in Iran, Afghanistan, and some Central Asian states. This has been seen that the Pashtun and Balochi speaking populations (Pathan and Baloch) showed more genetic affinity with the Central Asian, Afghan, and Iranian populations.

## Conclusion

The human Y-chromosome can be used for studying Y-STR haplotypes and determining their haplogroups which ultimately lead us to the ancient geographic origins of the studied population/individuals. In this study, allele frequencies and forensic parameters of the four Pakistani ethnic groups (Balochi, Punjabi, Pathan, and Sindhi) were calculated. These four groups and 83 regional ethnic groups were analyzed, and their corresponding haplotypes were compared. Using Y-STRs and available information of haplogroups from the Y-DNA phylogenetic tree, the geographic origin was traced. Results of our study showed us that according to the genetic makeup of these four ethnic groups belong to at least thirteen specific haplogroups with thirteen different lines of ancestry and geographic origins. Above 84% of these ethnic groups belongs to only six different lines of ancestry and geographic origins. Overall, the 17 Yfiler STRs included in the Yfiler kit are slowly to moderate mutating and can be used in sexual assault cases, paternity casework involving male offspring, or missing person analysis. More studies on extended sets of STRs are required to better understand the genetic complexity of the Pakistani population. The recent inclusion of these data in the YHRD allows widespread use for forensic application and paternal population history reconstruction.

## Supplementary Information

**Additional file 1: Supplementary Table 1.** Raw genotypic data of 4 ethnic groups typed with Yfiler.

**Additional file 2: Supplementary Table 2.** Allele Frequencies and Forensic Parameters 4 ethnic groups.

**Additional file 3: Supplementary Table 3.** Pairwise Rst values (below diagonal) and their corresponding *p* values (above diagonal) between 4 ethnic groups and other reference Pakistani populations.

**Additional file 4: Supplementary Table 4.** Pairwise Fstvalues (below diagonal) and their corresponding *p* values (above diagonal) between 4 ethnic groups and other reference Pakistani populations.

**Additional file 5: Supplementary Table 5.** Pairwise Rst values (below diagonal) and their corresponding *p* values (above diagonal) between 4 ethnic groups and other reference Pakistani populations.

**Additional file 6: Supplementary Table 6.** Pairwise Fst values (below diagonal) and their corresponding *p* values (above diagonal) between 4 ethnic groups and other reference Pakistani populations.

## Data Availability

All data generated or analyzed during this study are included in this published article and its supplementary information files.
